# Effects of erlotinib therapy on [^11^C]erlotinib uptake in EGFR mutated, advanced NSCLC

**DOI:** 10.1186/s13550-016-0169-8

**Published:** 2016-02-09

**Authors:** Idris Bahce, Maqsood Yaqub, Hanane Errami, Robert C. Schuit, Patrick Schober, Erik Thunnissen, Albert D. Windhorst, Adriaan A. Lammertsma, Egbert F. Smit, N. Harry Hendrikse

**Affiliations:** Department of Pulmonary Diseases, VU University Medical Center, PO Box 7057, 1007MB Amsterdam, The Netherlands; Department of Radiology & Nuclear Medicine, VU University Medical Center, Amsterdam, The Netherlands; Department of Anesthesiology, VU University Medical Center, Amsterdam, The Netherlands; Department of Pathology, VU University Medical Center, Amsterdam, The Netherlands; Department of Clinical Pharmacology & Pharmacy, VU University Medical Center, Amsterdam, The Netherlands; Department of Thoracic Oncology, Netherlands Cancer Institute, Amsterdam, The Netherlands

**Keywords:** NSCLC, PET, [^11^C]Erlotinib, Erlotinib therapy

## Abstract

**Background:**

In non-small cell lung cancer (NSCLC) patients off erlotinib therapy, positron emission tomography (PET) using [^11^C]erlotinib distinguished epidermal growth factor receptor (EGFR) mutations from wild-type EGFR. However, tumor uptake of [^11^C]erlotinib during erlotinib therapy is unknown. Therefore, the aims of this study were to evaluate tumor [^11^C]erlotinib uptake in NSCLC patients both on and off erlotinib therapy, to evaluate the effect of erlotinib therapy on tumor perfusion and its correlation to tumor [^11^C]erlotinib uptake, and also, to investigate simplified uptake parameters using arterial and venous blood samples.

**Methods:**

Ten patients were to be scanned twice with a 1–2-week interval, i.e., on (E+) and off (E−) erlotinib therapy. Each procedure consisted of a low-dose CT scan, a 10-min dynamic [^15^O]H_2_O PET scan, and a 60-min dynamic [^11^C]erlotinib PET scan with arterial and venous sampling at six time points. In patients(E+), the optimal compartment model was analyzed using Akaike information criterion. In patients(E−), the uptake parameter was the volume of distribution (*V*_T_), estimated by using metabolite-corrected plasma input curves based on image-derived input functions and discrete arterial and venous blood samples. Tumor blood flow (TBF) was determined by rate constant of influx (K1) of [^15^O]H_2_O using the 1T2k model and correlated with *V*_T_ and K1 values of [^11^C]erlotinib. The investigated simplified parameters were standardized uptake value (SUV) and tumor-to-blood ratio (TBR) at 40–60 min pi interval.

**Results:**

Of the 13 patients included, ten were scanned twice. In patients(E+), [^11^C]erlotinib best fitted the 2T4k model with *V*_T_. In all patients, tumor *V*_T_(E+) was lower than *V*_T_(E−) (median *V*_T_(E−) = 1.61, range 0.77–3.01; median *V*_T_(E+) = 1.17, range 0.53–1.74; *P* = 0.004). Using [^15^O]H_2_O, five patients were scanned twice. TBF did not change with erlotinib therapy, TBF showed a positive trend towards correlation with [^11^C]erlotinib K1, but not with *V*_T_. TBR_40–50_ and TBR_50–60_, using both arterial and venous sampling, correlated with *V*_T_(E−) (all *r*_s_ >0.9, *P* < 0.001), while SUV did not. In patients off and on therapy, venous TBR underestimated arterial TBR by 26 ± 12 and 9 ± 9 %, respectively.

**Conclusions:**

In patients on erlotinib in therapeutic dose, tumor *V*_T_ decreases with high variability, independent of tumor perfusion. For simplification of [^11^C]erlotinib PET scanning protocols, both arterial and venous TBR 40–60 min post injection can be used; however, arterial and venous TBR values should not be interchanged as venous values underestimate arterial values.

**Trial registration:**

Registered at the Netherlands Trial Registry: NTR3670.

**Electronic supplementary material:**

The online version of this article (doi:10.1186/s13550-016-0169-8) contains supplementary material, which is available to authorized users.

## Background

Non-small cell lung cancer (NSCLC) therapy has entered an era of precision medicine with an ever-increasing amount of therapeutic agents directed against specific tumor targets. Positron emission tomography (PET) is sometimes used to study the pharmacokinetic behavior of these new agents and to identify patients that might be sensitive to these drugs [1]. For this, the molecularly targeting therapeutic agents are labeled with radionuclides to be used as PET tracers [2].

An important actionable target in NSCLC is the epidermal growth factor receptor (EGFR). EGFR tyrosine kinase inhibitors (TKIs), such as erlotinib, inhibit growth in tumors that thrive mainly on the EGFR pathway, such as tumors with an activating EGFR mutation (EGFRmut) [3, 4]. PET using carbon-11-labeled erlotinib, [^11^C]erlotinib, allowed to visualize and quantify tumor [^11^C]erlotinib uptake in patients with EGFR-mutated NSCLC [1, 5, 6]. Moreover, tumor [^11^C]erlotinib uptake was shown to be higher in TKI-sensitive EGFRmut tumors as compared to tumors with a wild-type EGFR, indicating that PET and [^11^C]erlotinib may identify patients that are sensitive to erlotinib therapy [1].

Typically, EGFR TKI achieves a median progression-free survival of approximately 9–10 months in the first line setting [7]. Ultimately, all EGFRmut patients develop resistance to EGFR TKI during the course of treatment. Various patterns of disease progression may be observed. In many patients, only a few tumor lesions will grow, while others remain unchanged. In such oligo-progressive cases, it is unclear whether EGFR TKI therapy should be discontinued [8]. Decision management could be guided by knowledge of the residual EGFR TKI sensitivity of the tumor lesions.

PET using [^11^C]erlotinib may provide a means to determine residual TKI uptake after disease progression appears, and consequently, may aid in deciding whether or not to discontinue EGFR TKI therapy. To answer this question, PET should preferably be performed during erlotinib treatment. However, thus far, [^11^C]erlotinib PET scans were performed exclusively in the absence of erlotinib exposure [1, 5, 6]. To be able to interpret [^11^C]erlotinib PET data of patients in this clinical setting, the effects caused by the presence of therapeutic concentrations of non-labeled erlotinib on tumor [^11^C]erlotinib uptake need to be investigated first. From a tracer pharmacokinetic perspective, studying the effects of erlotinib therapy on [^11^C]erlotinib metabolism, plasma concentration, and tumor uptake can improve our understanding of the tracer uptake. These pharmacokinetic insights are needed for optimization of scanning protocols and design of future TKI PET studies. At present, the literature concerning this topic is limited, and to the best of our knowledge, no other clinical trial scanned patients with a radiolabeled EGFR TKI during treatment with the same EGFR TKI.

We performed a pilot study to assess the effect of pharmacological erlotinib concentrations on tumor [^11^C]erlotinib uptake. The primary objective was to compare tumor tracer uptake using the gold standard measure for [^11^C]erlotinib uptake volume of distribution (*V*_T_) in the presence and absence of pharmacological concentrations of erlotinib. The secondary aim of this study was to assess the effects of erlotinib therapy on tumor blood flow and its correlation with tumor [^11^C]erlotinib uptake. In addition, for simplification of future protocols, we also investigated simplified uptake parameters, i.e., standardized uptake values (SUVs) and tumor-to-blood ratio (TBR). TBR was based on arterial samples; however, we also used venous samples. This was again important for simplification of future protocols as arterial cannulation could be omitted if venous sampling was proven to be a valid substitute.

## Methods

### Patients

Patients with histologically proven EGFRmut NSCLC who were either planned to initiate erlotinib therapy or to stop erlotinib therapy due to disease progression while on erlotinib were asked to participate. Key inclusion criteria were age above 18 years, life expectancy of at least 12 weeks, at least one tumor lesion with a diameter of at least 1.5 cm in the chest region as measured by CT, Karnofsky index >60 %, and a written informed consent. Exclusion criteria were claustrophobia, pregnancy or lactating patients, metal implants in the thorax (e.g., pacemakers) that could interfere with PET/CT imaging, and concurrent treatment with experimental drugs. The study was approved by the Medical Ethics Review Committee of the VU University Medical Center. All patients provided written informed consent prior to inclusion.

### Study design

The aim was to include ten patients with EGFRmut NSCLC, who underwent two PET scan sessions. Patients who were on erlotinib therapy (E+) stopped therapy on the day of their first scan. Patients who were erlotinib naïve (E−) started therapy, immediately following the scanning procedure on day 1. For all patients, a second PET scan session was performed after 7 to 14 days. All PET scans were planned to start at the same time of the day, i.e., at 1:00 p.m. Patients on erlotinib therapy were asked to take their last medication, i.e., erlotinib 150 mg, at 8:00 a.m.

### PET/CT scanning procedure

One cannula was inserted into the radial artery for arterial blood sampling and another one into a contralateral arm vein for tracer injection and venous blood sampling. Scans were performed on a Gemini TF-64 PET/CT scanner (Philips Medical Systems, Best, the Netherlands), which is a high performance, time-of-flight (TOF), fully three-dimensional PET scanner combined with a 64-slice Brilliance CT scanner [9]. First, 370-MBq [^15^O]H_2_O was injected intravenously, simultaneously starting a 10-min emission scan. Next, a low-dose CT scan (30 mAs, without contrast) was performed for attenuation correction. Subsequently, 349 ± 46 MBq of [^11^C]erlotinib (synthesized as previously described and corresponding to a non-pharmacological dose of approximately 16.2 μg “cold” erlotinib with ≥18.5 GBq/μmol specific activity) was injected intravenously, simultaneously starting a 60-min emission scan [1]. [^15^O]H_2_O and [^11^C]erlotinib emission scans were acquired in list-mode and reconstructed into 26 frames with progressive increase in frame duration (1 × 10, 8 × 5, 4 × 10, 2 × 15, 3 × 20, 2 × 30, and 6 × 60 s) and 36 frames (1 × 10, 8 × 5, 4 × 10, 2 × 15, 3 × 20, 2 × 30, 6 × 60, 4 × 150, 4 × 300, and 2 × 600 s), respectively. All appropriate corrections were applied for dead time, decay, random, scatter, and attenuation. Reconstruction of PET data was performed using the 3D row-action maximum-likelihood algorithm (RAMLA) with CT-based attenuation correction. The final voxel size was 4 × 4 × 4 mm^3^ and the spatial resolution 5–7 mm full-width at half-maximum. No corrections for patient motion were applied.

Arterial and venous samples (7 mL) were taken at six time points (i.e., at 5, 10, 20, 30, 40, and 60 min) after injection of [^11^C]erlotinib. For both arterial and venous samples, plasma polar [^11^C]erlotinib metabolites and whole blood and plasma radioactivity concentrations were measured, as described previously [1].

### Data analysis

For each patient, the primary tumor was identified on the low-dose CT scan, and the tumor contours were delineated visually at the margins of the tumor on all planes where the primary tumor was visible, to generate a three-dimensional tumor volume of interest (VOI). Large blood vessels and the liver were avoided as much as possible. We did not delineate tumors on PET, as [^11^C]erlotinib PET uptake depends strongly on tumor characteristics (e.g., EGFR mutation). CT-based contour delineation was performed using an in-house software, developed within the interactive data language (IDL Virtual Machine 6.2, RSI Inc., Boulder, CO, USA) environment. Then, tumor VOIs were projected onto the dynamic [^11^C]erlotinib PET scan to generate tumor [^11^C]erlotinib time activity curves (TACs). In addition, metabolite-corrected image-derived plasma input functions (IDIFs) were derived from VOIs drawn on ten subsequent slices within the descending aorta (approximately 7 mL). Then, this arterial whole blood TAC was calibrated by the whole blood activity concentrations measured from the six manually drawn arterial blood samples. Next, the data was multiplied by the multi-exponential function that best fitted the plasma-to-whole blood ratios, again derived from the manual samples, to generate a plasma TAC. Then, the plasma TAC was corrected for metabolites using a sigmoid function derived from the best fit to the measured parent fractions of the arterial samples. Finally, a correction for delay was applied; this metabolite-corrected plasma TAC was used as IDIF [10–12].

A distinction was made in processing the kinetic data from patients on and off therapy. Previously, in patients off therapy, the optimal model for tumor [^11^C]erlotinib pharmacokinetics was found to be the reversible two-tissue model (2T4k) [1]. In patients off therapy, all tumor TACs were analyzed using this model. It was unknown, however, whether the same model was also valid for patients on erlotinib therapy. Therefore, in the latter patients, first, the optimal model was identified by fitting tumor [^11^C]erlotinib TACs to three conventional compartment models (i.e., single tissue, irreversible two-tissue, and reversible two-tissue) [10]. Subsequently, the optimal model was chosen on the basis of the Akaike information criterion [13]. After establishing the optimal model for patients on erlotinib therapy, all tumor TACs were analyzed using the corresponding preferred model. Pharmacokinetic analysis and modeling of tumor TACs and IDIF was performed using in-house software, developed within MATLAB (MathWorks, Inc).

In order to understand the effects of metabolism on *V*_T_ values under erlotinib therapy, the change in tumor *V*_T_ was correlated with the level of metabolism. As the level of metabolism, the parent fraction measured at 60 min post injection was used.

For [^15^O]H_2_O, same VOIs were drawn on the CT scans accompanying the [^15^O]H_2_O PET scans, and then projected onto the [^15^O]H_2_O data. All tumor TACs were analyzed with the standard single tissue compartment model (1T2k) for [^15^O]H_2_O [14], resulting in estimates of tumor blood flow (TBF) as calculated by rate constant of influx (K1) of [^15^O]H_2_O [15].

### Simplified analyses

Accuracies of several simplified static approaches were evaluated. SUVs, normalized for patient weight and injected dose, were evaluated in the interval 40–50 and 50–60 min. In addition, TBR values were evaluated using both arterial and venous whole blood activity concentrations in the time interval 40–50 and 50–60 min. These intervals were chosen, as unpublished analysis of previous scans showed that TBR using whole blood activity between 40 and 60 min correlated best with *V*_T_ [1].

### Statistical analysis

Statistical analysis was performed using SPSS software (SPSS for Windows 20.0, SPSS, Inc., Chicago, USA) and GraphPad (GraphPad Prism version 5.00 for Windows, GraphPad Software, San Diego, CA, USA). Spearman’s correlation coefficient (*r*_s_) and simple linear regression were used for correlations. A two-tailed probability value of *P* < 0.05 was considered to be significant. Bland-Altman analysis was performed to assess agreement between venous TBR and arterial TBR, between TBR_40–50_ and TBR_50–60_, and between venous and arterial tracer parent fractions. The Wilcoxon matched-pairs signed-rank test was used to test differences between scans with and without erlotinib therapy regarding *V*_T_ values, parent fraction values, whole blood SUVs, and TBF values. This test was also used to assess differences between whole blood SUVs obtained with arterial and venous samples.

## Results

### Patient characteristics

Patient characteristics are shown in Table 1. As three patients could only be scanned once, a total of 13 patients were recruited in order to obtain ten patients who were scanned twice using [^11^C]erlotinib. In nine out of these ten patients, both quantitative kinetic analyses could be obtained. In the remaining patient, this was not possible due to technical problems with blood sampling. In seven out of nine evaluable patients, the first [^11^C]erlotinib PET scan was without erlotinib therapy. In the remaining two out of nine patients, the first scan was performed while receiving erlotinib therapy. In only five out of nine evaluable patients, both [^15^O]H_2_O TBF(E−) and TBF(E+) could be derived. In the remaining patients, data from both [^15^O]H_2_O PET scans could not be performed or analyzed due to technical problems, as indicated in Table 1.

**Table 1:** Patient characteristics

Number	Gender, age (years)	EGFR mutation (TKI sensitivity)	Response to erlotinib^a^	[^11^C]Erlotinib PET scans	Condition	Arterial sampling	Venous sampling	[^15^O]H_2_O PET scans	Remarks
			Change in *V*_T_^b^	Time interval to second scan (days)					
1	F	exon19 (p.delE746-A750) and exon20 (T790M)	PD	First scan	E+	Yes	Yes	Yes	
	60	(sensitive + resistant)	–	Second scan	E−	NA	NA	NA	Synthesis of [^11^C]erlotinib failed quality check
				7					
2	F	exon19 (p.delE746-A750)	CR	First scan	E−	Yes	Yes	Yes	
	82	(sensitive)	−52 %	Second scan	E+	Yes	NA	Yes	No venous sampling due to clogging of the venous cannula
				14					
3	M	exon18 (p.G719S and p.E709A)	PR	First scan	E−	Yes	Yes	Yes	
	74	(sensitive [22, 23])	−17 %	Second scan	E+	Yes	Yes	NA	Synthesis of [^15^O]H_2_O failed quality check
				13					
4	M	exon21 (p.P848L)	–	First scan	E−	Yes	Yes	Yes	
	66	(resistant [24])	−43 %	Second scan	E+	Yes	Yes	Yes	
				7					
5	F	exon19 (p.delE746-T751) (sensitive)	PD	First scan	E+	Yes	Yes	NA	Synthesis of [^15^O]H_2_O failed quality check
	61		–	Second scan	NA	NA	NA	NA	Yield of [^11^C]erlotinib synthesis too low
				7					
6	F	exon19 (p.delE746-A750) and exon20 (p.T790M)	Slow PD	First scan	E+	Yes	Yes	Yes	
	45	(sensitive + resistant)	−47 %	Second scan	E−	Yes	Yes	Yes	
				10					
7	M	exon19 (p.E746-S752) and exon20 (p.T790M)	SD	First scan	E−	NA	NA	NA	Aberrant arterial and venous blood sample values
	74	(sensitive + resistant)	–						Aberrant [^15^O]H_2_O PET data
				Second scan	E+	Yes	Yes	Yes	
				7					
8	M	exon18 (p.G719S) and exon20 (p.S768I)	SD	First scan	E−	Yes	Yes	Yes	
	81	(unclear [25, 26, 27])	−58 %	Second scan	E+	Yes	Yes	Yes	
				7					
9	F	exon19 (p.delE746-A750)	PR	First scan	E−	Yes	Yes	Yes	Aberrant [^15^O]H_2_O PET data
	55	(sensitive)	−29 %	Second scan	E+	Yes	Yes	Yes	
				10					
10	F	exon19 (p.delL747-S752 and p.P753Q) and exon20 (p.T790M)	Slow PD	First scan	E+	Yes	Yes	NA	No [^15^O]H_2_O synthesis
	71	(sensitive + resistant)	−25 %	Second scan	E−	Yes	Yes	NA	No [^15^O]H_2_O synthesis
				14					
11	F	exon21 (p.L861Q)	SD	First scan	E−	Yes	Yes	Yes	
	77	(sensitive [28])	−39 %	Second scan	E+	Yes	Yes	NA	No [^15^O]H_2_O synthesis
				7					
12	M	exon19 (p.delE746 S752)	PR	First scan	E−	Yes	Yes	Yes	
	70	(sensitive)	–	Second scan	NA	NA	NA	NA	Yield of [^11^C]erlotinib synthesis too low
				7					
13	F	exon21 (p.L858R) and exon20 (p.T790M)	Slow PD	First scan	E−	Yes	Yes	Yes	
	74	(sensitive + resistant)	−40 %	Second scan	E+	Yes	Yes	Yes	
				7					

*Abbreviations: F* female, *M* male, *E+* with erlotinib therapy, *E−* without erlotinib therapy, *CR* complete response, *PR* partial response, *SD* stable disease, *PD* progressive disease, *NA* not available

^a^Tumor response to erlotinib as evaluated at the time of the first scan (in patients stopping erlotinib) or tumor response to erlotinib after its initiation (in patients starting erlotinib therapy)

^b^Change in *V*_T_ as defined by (*V*_T_(E+) − *V*_T_(E−)) / *V*_T_(E−)

### Effects of erlotinib therapy on plasma kinetics

**Fig. 1:**
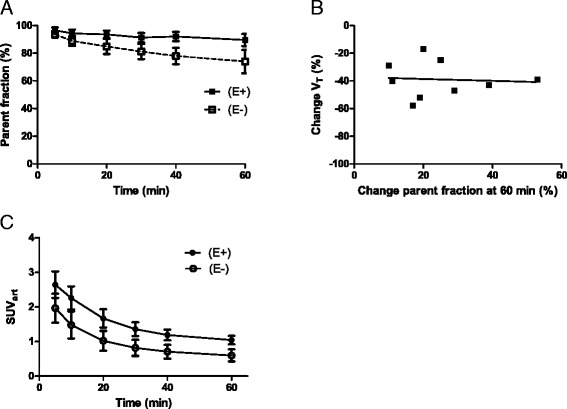
Effect of erlotinib therapy on parent fractions. Comparison of mean (±SD) arterial parent fractions of [^11^C]erlotinib with and without erlotinib therapy **(a)**. Correlation of *V*_T_ change and arterial parent fractions change at 60 min post injection **(b)**. Arterial blood SUV, i.e., activity normalized to injected dose and patient weight, in patients off and on therapy **(c)**. Abbreviations: *V*_*T*_ volume of distribution, *SUV* standardized uptake value, *E−* without erlotinib therapy, *E+* with erlotinib therapy

Parent fractions of [^11^C]erlotinib, as measured in arterial plasma samples, were higher in patients on therapy at all time points (all *P* values <0.05, see Fig. 1a). Change in tracer metabolism during erlotinib therapy did not correlate with changes in *V*_T_ (*r*_s_ = 0.33, *P* = 0.385), as shown in Fig. 1b.

Arterial blood activity, normalized to injected dose and patient weight, was also higher in patients on therapy at all time points (all *P* values <0.05, Fig. 1c). Detailed results are shown in Additional file 1: Tables S1 and S2.

### Effects of erlotinib therapy on kinetic modeling and tumor [^11^C]erlotinib uptake

**Fig. 2:**
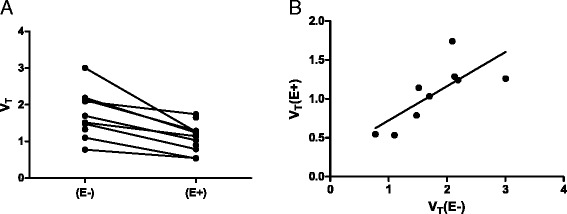
Tumor *V*_T_ values. *V*_T_ values of all patients without and with erlotinib therapy **(a)**. Correlation between *V*_T_ with and *V*_T_ without erlotinib therapy **(b)**. Abbreviations: *V*_*T*_ volume of distribution, *E−* without erlotinib therapy, *E+* with erlotinib therapy

According to the Akaike information criterion, the reversible two-tissue compartment model (2T4k) was the preferred model in all patients(E+). This was also the case for patients(E−), confirming previous findings [1, 5, 6]. In all nine evaluable patients, tumor [^11^C]erlotinib *V*_T_(E+) was significantly lower than *V*_T_(E−) with a mean (±SD) intrapatient decrease of 38 ± 13 % (median *V*_T_(E−) = 1.61, range 0.77–3.01; median *V*_T_(E+) = 1.17, range 0.53–1.74; *P* = 0.004; see Fig. 2a). There was a good correlation between *V*_T_(E+) and *V*_T_(E−) (*r*_s_ = 0.82; *P* = 0.011), as shown in Fig. 2b. See Additional file 1: Table S3 for detailed results.

### Effects of erlotinib therapy on tumor [^15^O]H_2_O perfusion

**Fig. 3:**
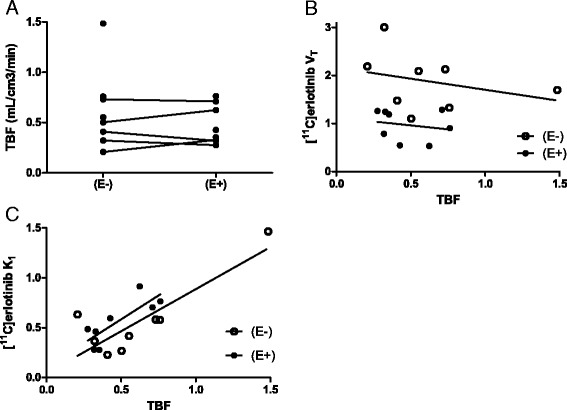
Tumor blood flow. TBF values of patients without (*N* = 8) and with (*N* = 8) erlotinib therapy **(a)**. Correlation of tumor perfusion (TBF) with *V*_T_ **(b)** and [^11^C]erlotinib K1 **(c)**. Abbreviations: *TBF* tumor blood flow, *V*_*T*_ volume of distribution, *E−* without erlotinib therapy, *E+* with erlotinib therapy, *K1* influx rate constant

Tumor [^15^O]H_2_O perfusion did not change between patients on (*N* = 8) and off (*N* = 8) erlotinib therapy (with a mean ± SD TBF of 0.475 ± 0.194 and 0.622 ± 0.397 mL/cm^3^/min, *P* = 0.813, respectively, see Fig. 3a). There was no correlation between TBF and [^11^C]erlotinib *V*_T_ in patients(E−) and patients(E+) (*r*_s_ = −0.452, *P* = 0.268 and *r*_s_ = −0.167, *P* = 0.703, respectively, see Fig. 3b). Tumor rate constant of [^11^C]erlotinib influx, i.e., K1, showed a trend towards positive correlation with TBF(E−) and TBF(E+); however, this was not statistically significant (*r*_s_ = 0.714, *P* = 0.058 and *r*_s_ = 0.405, *P* = 0.327, respectively, see Fig. 3c) (see Additional file 1: Table S4 for detailed results).

### Effects of erlotinib therapy on simplified uptake parameters

**Fig. 4:**
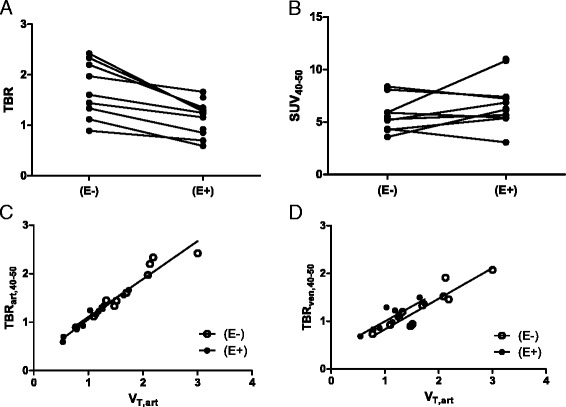
Simplified uptake parameters. TBR values from the 40–50-min post injection interval, using arterial samples **(a)** and SUVs from the 40–50-min post injection interval **(b)** in all patients off and on erlotinib therapy. Correlation between TBR values from the 40–50-min post injection interval, using arterial **(c)** and venous **(d)** samples, with *V*_T_ in all patients off and on erlotinib therapy. Abbreviations: *TBR* tumor-to-blood ratio, *SUV* standardized uptake value, *V*_*T*_ volume of distribution, *E−* without erlotinib therapy, *E+* with erlotinib therapy

SUVs did not correlate with *V*_T_ values, both on and off erlotinib therapy (*r*_s_ = 0.39, *P* = 0.260 and *r*_s_ = 0.30, *P* = 0.342, respectively). However, TBR_40–50_ values showed good correlation with *V*_T_ values, both on and off therapy (*r*_s_ = 0.97, *P* < 0.001 and *r*_s_ = 0.96, *P* < 0.001, respectively), as shown in Fig. 4. TBR_50–60_ also showed good correlation (with *r*_s_ = 0.92, *P* < 0.001 and *r*_s_ = 0.99, *P* < 0.001, respectively). The mean (±SD) difference between TBR_40–50_ and TBR_50–60_ in patients off therapy was 4 ± 7 % and 1 ± 7 % in patients on therapy.

**Fig. 5:**
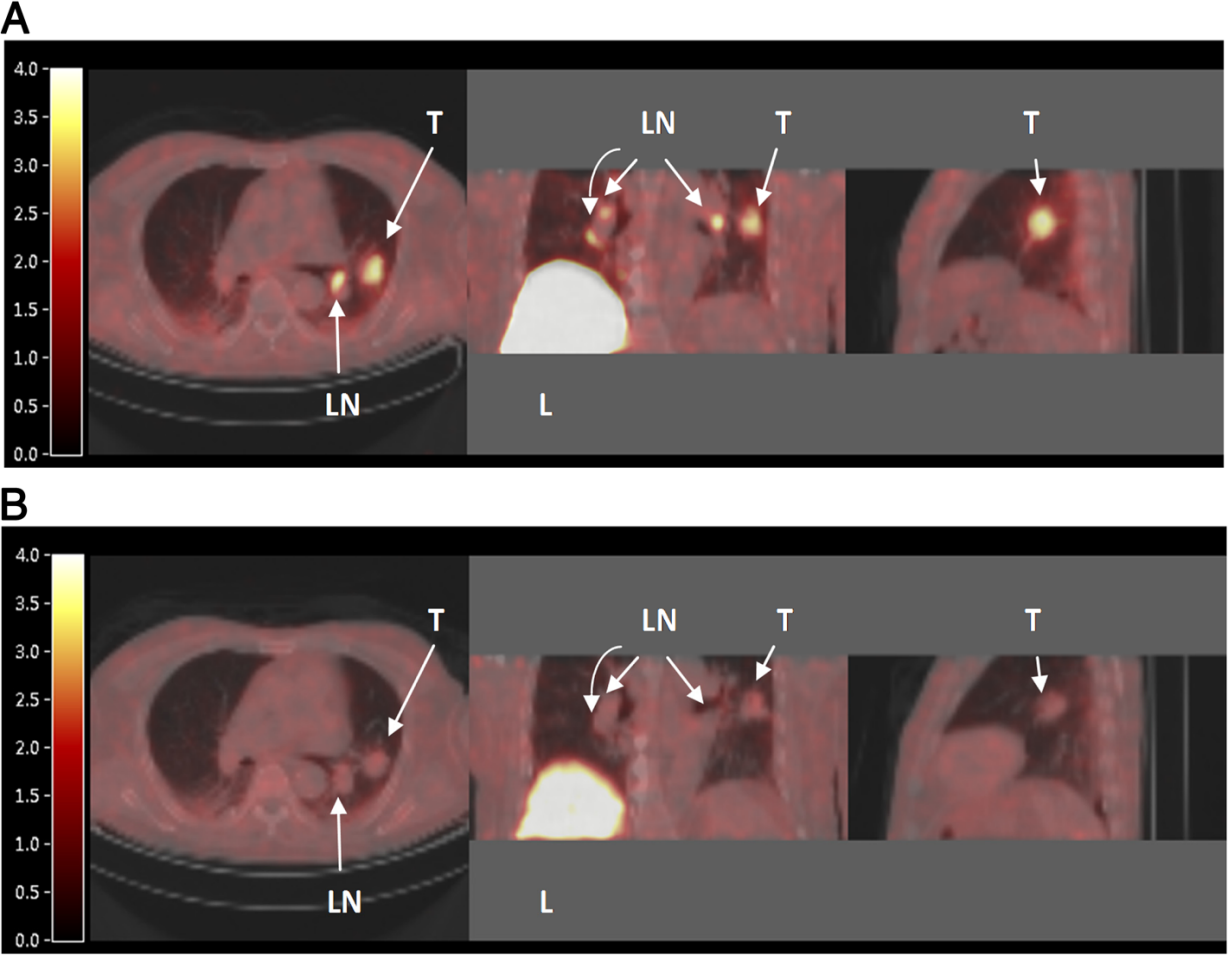
[^11^C]Erlotinib PET images. PET images of a typical patient (Nr 8), who was scanned first off erlotinib therapy **(a)**; he then started therapy and was scanned again after 7 days **(b)**. Axial, coronal, and sagittal views are shown, obtained by CT-fused parametrically reconstructed [^11^C]erlotinib TBR_art,50–60_ PET images. The color scale indicates the TBR_art,50–60_ value per pixel (unitless). The primary tumor lesion (*T*) and regional lymph nodes (*LN*) are clearly visible in the absence of erlotinib therapy. Also, high uptake is seen in the liver (*L*)

Representative parametric [^11^C]erlotinib images, using TBR_50–60_, of a typical patient off and on erlotinib therapy are shown in Fig. 5.

#### Arterial versus venous whole blood activity

**Fig. 6:**
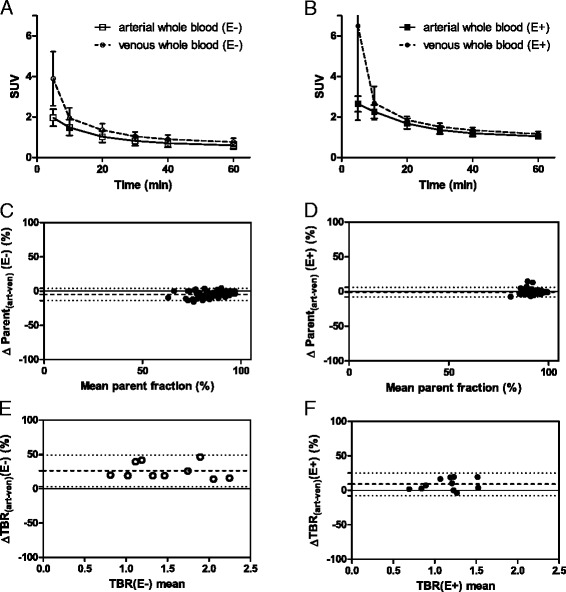
Arterial and venous sampling. Whole blood SUVs (mean ± SD) obtained from venous and arterial samples, in patients off **(a)** and on **(b)** erlotinib therapy. Bland-Altman plots demonstrating the arterial-venous parent fraction difference (%) per mean parent fraction value in patients off **(c)** and on **(d)** erlotinib therapy. In patients(E−) and patients(E+), a bias of −4.8 ± 4.6 and −0.7 ± 3.5 % was seen, respectively. *Dotted lines* indicate the mean bias and the 95 % limits of agreement. Next, Bland-Altman plots demonstrating the arterial-venous TBR_40–50_ difference (%) per mean *V*_T_ value, in ten patients(E−) **(e)** and 11 patients(E+) **(f)** that had evaluable arterial and venous TBR values. The *horizontal line* indicates zero difference between arterial and venous TBR_40–50_ measures, values above the zero difference line indicate lower venous TBR_40–50_ values (i.e., an underestimation). In patients(E−) and patients(E+), a bias (i.e., average of the differences) of 26 ± 12 and 9 ± 9 % was seen, respectively. *Dotted lines* indicate the mean bias and the 95 % limits of agreement. Abbreviations: *TBR* tumor-to-blood ratio, *SUV* standardized uptake value, *E−* without erlotinib therapy, *E+* with erlotinib therapy

The mean venous blood activity values, normalized to injected dose and patient weight, were higher than the mean arterial values for all measured time points in patients(E−) (*P* < 0.05). In patients(E+) at 5 min post injection, the venous values were significantly higher than arterial values, and at the remaining time points, no difference was observed. Figure 6a, b illustrates the mean ± SD whole blood SUV obtained from venous and arterial samples, in patients off and on erlotinib therapy.

#### Arterial versus venous metabolites

Metabolite analyses showed a good correlation between arterial and venous samples (*r*_s_ = 0.91, *P* < 0.001 and *r*_s_ = 0.76, *P* < 0.001) in patients(E−) and patients(E+), respectively. There was a good agreement between arterial and venous parent fractions, both off and on erlotinib therapy (with an average (±SD) bias of −4.8 ± 4.6 and −0.7 ± 3.5 %, respectively, see Fig. 6c, d).

#### Arterial versus venous TBR

Only eight out of nine evaluable patients had arterial and venous sampling in both conditions, i.e., with and without erlotinib therapy. TBR_40–50_ values obtained using arterial and venous blood samples showed good correlation with each other, both off (*r*_s_ = 0.95, *P* < 0.001) and on (*r*_s_ = 0.83, *P* = 0.002) erlotinib therapy. Similar results were obtained with TBR_50–60_ (with *r*_s_(E−) = 0.89, *P* < 0.001 and *r*_s_(E+) = 0.93, *P* < 0.001).

Venous sampling underestimated TBR_40–50_ as compared to arterial sampling in patients(E−) and patients(E+) by an average (±SD) of 26 ± 12 and 9 ± 9 %, respectively (see Fig. 6e, f). However, venous TBR did have a good correlation with arterial *V*_T_ (*r*_s_(E−) = 0.90, *P* < 0.001 and *r*_s_(E+) = 0.79, *P* = 0.006), as shown in Fig. 4d (see Additional file 1: Table S5 for individual results).

## Discussion

### Effects of erlotinib therapy on tumor tracer uptake

The present study demonstrated that tumor [^11^C]erlotinib *V*_T_ decreases significantly during erlotinib therapy.

To our best knowledge, this is the first clinical study that investigated the change of radiolabeled EGFR TKI uptake in patients off and on treatment using the same EGFR TKI. In the presence of therapeutic concentrations of erlotinib, tumor [^11^C]erlotinib uptake decreased. This was presumably caused by occupancy of EGF receptors by abundantly present non-labeled erlotinib, i.e., due to a decrease in available binding sites. Blocking studies in xenograft models provide support for this mechanism. Using [^11^C]erlotinib, Petrulli et al. showed that NSCLC xenografts with activating EGFRmut (HCC827) in mice had lower tracer uptake when cold erlotinib was given along with the tracer [16]. In addition, Abourbeh et al. showed in mice-bearing HCC827 xenografts more than 50 % reduction in tumor [^11^C]erlotinib uptake after administration of excess non-labeled erlotinib [17]. Similar results were obtained with other radiolabeled TKI, such as [^18^F]afatinib [18] and [^11^C]PD153035 [19]. The fact that there was consistent decrease in [^11^C]erlotinib uptake in the present study supports the notion that uptake of [^11^C]erlotinib is, at least in part, due to specific binding. Furthermore, in the presence of therapeutic concentrations of erlotinib, obtained by taking a fixed oral dose of 150 mg erlotinib daily, there was still residual tumor tracer uptake. Interestingly, from a pharmacokinetic perspective, this may indicate that there may be room for increasing the therapeutic concentration of erlotinib, as at maximal concentration, the specific binding would be absent.

Erlotinib therapy is known to induce metabolizing enzymes, such as CYP1A, CYP3A4, and CYP3A5 [20]. Also, in vitro data suggest that erlotinib stimulates the metabolism of midazolam in human microsomes, suggesting that erlotinib could induce its own metabolism and thus also increase the clearance of [^11^C]erlotinib [21]. However, this was not observed in the present study. On the contrary, parent fractions at 60 min post injection were significantly higher during erlotinib therapy. Possibly, the presence of abundant non-labeled erlotinib also saturated the metabolizing enzymes, thereby slowing down the metabolism of [^11^C]erlotinib. Moreover, patients on therapy had higher blood activity concentrations, normalized to injected dose and patient weight. This may also be caused by higher concentrations of circulating parent tracer due to the blocking of receptors and enzymes by high concentrations of non-labeled erlotinib.

Among the nine evaluable patients, two were scanned first under erlotinib therapy and stopped therapy immediately thereafter. In these two patients, the abovementioned findings were also true, i.e., *V*_T_(E+) was lower than *V*_T_(E−) and metabolites(E+) were lower than metabolites(E−). This supports the notion that the presence of non-labeled erlotinib determined these pharmacokinetic changes by the abovementioned mechanism.

High tumor sensitivity to erlotinib could potentially cause a large decrease in *V*_T_. Namely, in the absence of cold erlotinib, EGFR-TKI-sensitive tumors are expected to have high [^11^C]erlotinib *V*_T_ values as compared to resistant tumors [1]. Once cold erlotinib is added, EGF receptors become blocked causing *V*_T_ to drop. The results of this study confirmed that the patients with the largest decrease in *V*_T_ did have responsive tumors; however, there was no clear association between decrease in *V*_T_ and tumor response. To illustrate, three patients (patients 8, 2, and 6) had a large (i.e., approximately 50 %) decrease in *V*_T_. Patient 8 was treated with erlotinib therapy for a few weeks only. Erlotinib was stopped, as he refused to continue therapy due to a pneumonia that he ascribed to erlotinib. He did have some tumor regression with erlotinib during these few weeks, however, not enough to be declared a partial response. Patient 2 had a complete response to erlotinib therapy after 3 months. Patient 6 had a slow disease progression prior to erlotinib scanning; she stopped erlotinib therapy after her first scan but developed a severe flare of her disease within 1 week. Her second scan showed increased tumor volume and increased *V*_T_; this illustrates that her tumor still had significant amount of sensitive clones. These cases demonstrate that high decrease in *V*_T_ can occur in sensitive tumors; however, there was no clear association, that is, responders did not exclusively show high decrease, as there were two other cases with partial tumor response to erlotinib therapy who showed moderate decrease in *V*_T_ of 29 and 17 %. On the other hand, as a result of erlotinib therapy, changes can occur in the size of the tumor, its concentration of vital tumor cells and possibly its EGFR density. These changes can occur in a period as short as 7 to 14 days after initiation or discontinuation of therapy and may also influence *V*_T_. Therefore, any tumor response to erlotinib therapy may also influence the decrease of *V*_T_ during therapy. However, the limited number of patients scanned does not allow for extensive elaboration. Future studies including more patients should investigate the correlation between response and change in uptake.

The decrease in *V*_T_ varied between 17 and 58 %. This high level of variability disqualifies *V*_T_(E+) as substitute for *V*_T_(E−). Any quantitative comparison between patients or between different time points in a single patient should be performed using *V*_T_(E−). However, for intrapatient interlesional comparison at a single time point, *V*_T_(E+) may still be considered. Whether tumor TKI sensitivity can be predicted by [^11^C]erlotinib *V*_T_(E+) remains to be investigated.

### Effect of erlotinib therapy on tumor perfusion

Tumor perfusion was not changed by erlotinib treatment. Also, tumor perfusion showed no association with [^11^C]erlotinib *V*_T_(E−) nor with *V*_T_(E+). However, there was a positive trend between tumor perfusion and the delivery of [^11^C]erlotinib to the tumor, which is in accordance with the 2T4k model. These findings suggest that the extraction of [^11^C]erlotinib remains unchanged during erlotinib therapy.

### Simplified uptake parameters

For simplification of future scanning protocols, SUV was not found to be a suitable uptake parameter, as it did not correlate with *V*_T,_ both on and off therapy. SUV normalizes on the basis of injected dose and patient weight, which is less accurate as compared to TBR that normalizes on the basis of the blood pool activity itself. For example, during erlotinib therapy, the blood tracer concentrations were higher. Due to this increased tracer availability, the absolute amount of tumor tracer binding may have changed in varying extent. Tumor SUV does not take this variable into account, whereas TBR does. Contrary to SUV, arterial and venous TBR showed an excellent correlation with arterial *V*_T_, supporting the use of both arterial and venous TBR in the time interval of 40 to 60 min post injection in future whole body static scanning protocols.

Interestingly, venous TBR values were lower than the arterial TBR values, especially in patients off therapy. This was due to higher plasma activity in venous samples than in arterial samples. As no difference in metabolism was observed between venous and arterial samples, the higher venous plasma activity values were only caused by a higher venous concentration of parent molecules. The reason for this finding is unclear. Possibly, the interstitial compartment together with EGFR molecules that are highly expressed at the epidermal tissue compartment act as a capacitator, by reversibly binding [^11^C]erlotinib molecules. So, venous plasma collects not only the unbound [^11^C]erlotinib molecules coming from the arterio-capillary route but also the [^11^C]erlotinib molecules being released from the interstitial and peripheral tissue compartments. This can also explain why patients on therapy, who have more EGFR saturation, have less veno-arterial activity difference. Another cause that may be considered is the fact that a single venous cannula was used for tracer injection and blood withdrawal, which implies that venous activity may increase due to the presence of tracer molecules that remained sticking to the cannula wall during injection. However, this mechanism is unlikely as it cannot explain why the veno-arterial difference was higher in off therapy than on therapy. Nevertheless, venous sampling was found to be suitable for interlesional quantitative comparison using TBR, as long as venous values are not interchanged together with arterial values.

### Limitations

This study was limited by the fact that not all uptake values of [^11^C]erlotinib and [^15^O]H_2_O and not all arterial and venous sampling values were present or evaluable in all patients; this was due to practical limitations as mentioned in Table 1. The small number of patients did not allow to establish the clinical role of [^11^C]erlotinib PET during erlotinib therapy. Larger studies are needed to explore the clinical benefits of scanning during therapy, e.g., for evaluating interlesional differences within a single patient, preferably using TBR as validated in the current study.

## Conclusions

Therapeutically dosed oral erlotinib decreases tumor [^11^C]erlotinib *V*_T_ with high variability, independent of tumor perfusion. In patients on erlotinib therapy, quantitative tracer uptake analysis using *V*_T_ does not seem appropriate; however, it may be useful for intrapatient comparison of tumor lesions, which remains to be investigated. For protocol simplification, both arterial and venous TBR, in the time interval between 40 and 60 min post injection, could be used; however, arterial and venous TBR values should not be interchanged as venous values underestimate arterial values.

## Additional file

Additional file 1:
**Supplementary data.** **Table S1.** Parent fractions (%). **Table S2.** Whole blood SUV. **Table S3.** Tumor [^11^C]erlotinib *V*_T_ and K1 values. **Table S4.** Tumor [^15^O]H_2_O flow values. Table S5. SUV and TBR values (unitless). (DOC 170 kb)

## Abbreviations

CT: computed tomography
E−: without erlotinib therapy
E+: under erlotinib therapy
EGFR: epidermal growth factor receptor
IDIF: image-derived input function
K1: rate constant of influx
NSCLC: non-small cell lung cancer
PET: positron emission tomography
*r*_s_: Spearman’s correlation coefficient
TAC: time activity curve
TBF: tumor blood flow
TKI: tyrosine kinase inhibitor
VOI: volume of interest
*V*_T_: volume of distribution
