# Supplementary material for: Effects of erlotinib therapy on [11C]erlotinib uptake in EGFR mutated, advanced NSCLC
Source: EJNMMI Res. 2016 Feb 9;6:10. doi: 10.1186/s13550-016-0169-8 (PMC4746207; doi:10.1186/s13550-016-0169-8)
Supplement: Additional file 1: — Supplementary data. Table S1. Parent fractions (%). Table S2. Whole blood SUV. Table S3. Tumor [11C]erlotinib V T and K1 values. Table S4. Tumor [15O]H2O flow values. Table S5. SUV and TBR values (unitless). (DOC 170 kb) [file 13550_2016_169_MOESM1_ESM.doc]

# Supplementary data

## Supplementary Table S1. Parent fractions (%)

| **Pt nr** | **Arterial samples** | | | | | | **Venous samples** | | | | | |
| --- | --- | --- | --- | --- | --- | --- | --- | --- | --- | --- | --- | --- |
|  | **E-** | | | | | | | | | | | |
|  | **5**  **min** | **10**  **min** | **20**  **min** | **30**  **min** | **40**  **min** | **60**  **min** | **5**  **min** | **10**  **min** | **20**  **min** | **30**  **min** | **40**  **min** | **60**  **min** |
| **1** | NA | NA | NA | NA | NA | NA | NA | NA | NA | NA | NA | NA |
| **2** | 93 | 88 | 83 | 80 | 80 | 76 | 95 | 92 | 88 | 86 | 84 | 79 |
| **3** | 95 | 93 | 86 | 86 | 83 | 77 | 97 | 93 | 89 | 88 | 85 | 80 |
| **4** | 93 | 88 | 84 | 79 | 76 | 71 | 91 | 83 | 77 | 74 | 73 | 66 |
| **5** | NA | NA | NA | NA | NA | NA | NA | NA | NA | NA | NA | NA |
| **6** | 95 | 91 | 87 | 79 | 75 | 68 | 99 | 97 | 92 | 89 | 86 | 78 |
| **7** | NA | NA | NA | NA | NA | NA | NA | NA | NA | NA | NA | NA |
| **8** | 96 | 90 | 85 | 83 | 78 | 78 | 96 | 94 | 88 | 80 | 80 | 76 |
| **9** | 89 | 90 | 92 | 85 | 81 | 82 | 87 | 92 | 88 | 87 | 89 | 85 |
| **10** | 95 | 88 | 84 | 81 | 76 | 70 | 98 | 96 | 93 | 89 | 84 | 82 |
| **11** | 93 | 84 | 76 | 73 | 68 | 60 | 93 | 90 | 82 | 82 | 76 | 66 |
| **12** | 95 | 95 | 92 | 90 | 89 | 87 | 97 | 95 | 95 | 93 | 92 | 90 |
| **13** | 97 | 92 | 89 | 88 | 84 | 82 | 97 | 94 | 94 | NA | 89 | 86 |
|  | **E+** | | | | | | | | | | | |
| **1** | 92 | 88 | 88 | 88 | 84 | 78 | 95 | 94 | 93 | 84 | 87 | 84 |
| **2** | 97 | 94 | 92 | 91 | 92 | 91 | 97 | 98 | NA | NA | NA | NA |
| **3** | 97 | 95 | 95 | 96 | 93 | 93 | 98 | 97 | 97 | 94 | 90 | 94 |
| **4** | 97 | 95 | 94 | 92 | 93 | 91 | 97 | 97 | 95 | 94 | 93 | 92 |
| **5** | 99 | 98 | 98 | 97 | 97 | 96 | 99 | 98 | 98 | 98 | 98 | 96 |
| **6** | 94 | 95 | 93 | 89 | 91 | 88 | 95 | 93 | 91 | 90 | 90 | 88 |
| **7** | 95 | 95 | 90 | 87 | 91 | 86 | 96 | 94 | 95 | 89 | 92 | 90 |
| **8** | 97 | 93 | 95 | 88 | 92 | 91 | 96 | 95 | 95 | 92 | 92 | 94 |
| **9** | 98 | 92 | 94 | 89 | 96 | 90 | 86 | 86 | 94 | 92 | 83 | 87 |
| **10** | 97 | 94 | 93 | 90 | 89 | 88 | 97 | 95 | 93 | 93 | 91 | 89 |
| **11** | 98 | 97 | 96 | 94 | 93 | 92 | 99 | 98 | 97 | 96 | 95 | 93 |
| **12** | NA | NA | NA | NA | NA | NA | NA | NA | NA | NA | NA | NA |
| **13** | 99 | 97 | 96 | 95 | 94 | 91 | 100 | 98 | 98 | 97 | 96 | 94 |

**Abbreviations:** (E+) = with erlotinib therapy, (E-) = without erlotinib therapy, NA = not available.

## Supplementary Table S2. Whole blood SUV (unitless)

| **Pt nr** | **Arterial samples** | | | | | | **Venous samples** | | | | | |
| --- | --- | --- | --- | --- | --- | --- | --- | --- | --- | --- | --- | --- |
|  | **E-** | | | | | | | | | | | |
|  | **5**  **min** | **10**  **min** | **20**  **min** | **30**  **min** | **40**  **min** | **60**  **min** | **5**  **min** | **10**  **min** | **20**  **min** | **30**  **min** | **40**  **min** | **60**  **min** |
| **1** | NA | NA | NA | NA | NA | NA | NA | NA | NA | NA | NA | NA |
| **2** | 1,58 | 1,13 | 0,82 | 0,72 | 0,66 | 0,59 | 2,60 | 1,38 | 1,03 | 0,88 | 0,81 | 0,69 |
| **3** | 1,87 | 1,51 | 1,06 | 0,86 | 0,75 | 0,62 | 4,83 | 2,12 | 1,43 | 1,11 | 0,87 | 0,76 |
| **4** | 1,59 | 0,85 | 0,57 | 0,43 | 0,36 | 0,29 | 1,87 | 1,45 | 0,97 | 0,76 | 0,61 | 0,47 |
| **5** | NA | NA | NA | NA | NA | NA | NA | NA | NA | NA | NA | NA |
| **6** | 1,56 | 1,32 | 0,91 | 0,71 | 0,63 | 0,54 | 5,50 | 2,43 | 1,35 | 0,99 | 0,84 | 0,72 |
| **7** | NA | NA | NA | NA | NA | NA | NA | NA | NA | NA | NA | NA |
| **8** | 1,79 | 1,41 | 0,99 | 0,80 | 0,71 | 0,64 | 2,49 | 1,73 | 1,23 | 0,93 | 0,81 | 0,69 |
| **9** | 2,84 | 2,24 | 1,59 | 1,27 | 1,08 | 0,89 | 5,62 | 2,88 | 1,95 | 1,51 | 1,28 | 1,07 |
| **10** | 1,73 | 1,38 | 0,96 | 0,77 | 0,65 | 0,53 | 4,47 | 2,46 | 1,60 | 1,08 | 0,87 | 0,78 |
| **11** | 2,09 | 1,37 | 0,82 | 0,62 | 0,52 | 0,41 | 4,61 | 1,44 | 0,99 | 0,82 | 0,65 | 0,51 |
| **12** | 2,45 | 1,87 | 1,28 | 1,04 | 0,91 | 0,79 | 4,01 | 1,84 | 1,63 | 1,21 | 1,20 | 1,03 |
| **13** | 2,14 | 1,73 | 1,23 | 0,96 | 0,82 | 0,69 | 2,88 | 1,71 | 1,32 | 1,13 | 1,02 | 0,88 |
|  | **E+** | | | | | | | | | | | |
| **1** | 2,79 | 2,37 | 1,67 | 1,28 | 1,11 | 0,99 | 5,60 | 2,36 | 1,70 | 1,42 | 1,30 | 1,06 |
| **2** | 2,16 | 1,63 | 1,17 | 1,04 | 0,98 | 0,96 | 18,53 | 5,06 | NA | NA | NA | NA |
| **3** | 2,53 | 2,19 | 1,62 | 1,32 | 1,17 | 0,99 | 5,73 | 2,98 | 1,97 | 1,54 | 1,33 | 1,15 |
| **4** | 2,24 | 2,00 | 1,53 | 1,21 | 1,09 | 0,93 | 4,08 | 2,62 | 1,83 | 1,44 | 1,27 | 1,11 |
| **5** | 3,37 | 2,96 | 2,27 | 1,75 | 1,44 | 1,19 | 2,89 | 2,73 | 2,21 | 1,81 | 1,57 | 1,34 |
| **6** | 2,61 | 2,22 | 1,61 | 1,32 | 1,14 | 0,97 | 4,58 | 2,18 | 1,75 | 1,35 | 1,17 | 1,02 |
| **7** | 2,72 | 2,32 | 1,74 | 1,44 | 1,27 | 1,13 | 3,09 | 1,92 | 1,77 | 1,54 | 1,41 | 1,22 |
| **8** | 2,34 | 2,16 | 1,64 | 1,34 | 1,16 | 1,00 | 4,27 | 2,53 | 1,82 | 1,43 | 1,27 | 1,09 |
| **9** | 3,31 | 2,71 | 1,99 | 1,70 | 1,52 | 1,35 | 5,22 | 2,66 | 1,98 | 1,77 | 1,55 | 1,37 |
| **10** | 2,32 | 2,14 | 1,69 | 1,39 | 1,22 | 1,04 | 8,12 | 2,74 | 1,93 | 1,50 | 1,42 | NA |
| **11** | 2,77 | 2,14 | 1,53 | 1,26 | 1,12 | 1,03 | 3,21 | 1,91 | 1,51 | 1,19 | 1,13 | 1,03 |
| **12** | NA | NA | NA | NA | NA | NA | NA | NA | NA | NA | NA | NA |
| **13** | 2,60 | 2,26 | 1,58 | 1,24 | 1,09 | 0,97 | 12,55 | 2,52 | 1,93 | 1,63 | 1,35 | 1,15 |

**Abbreviations:** (E+) = with erlotinib therapy, (E-) = without erlotinib therapy, NA = not available.

## Supplementary Table S3. Tumor [^11^C]erlotinib V_T_ and K1 values (unitless)

| **Pt Nr** | **Arterial samples** | | | |
| --- | --- | --- | --- | --- |
|  | **V_T_(E-)** | **SD V_T_(E-)** | **V_T_(E+)** | **SD V_T_(E+)** |
| **1** | NA | NA | 0,903 | 0,068 |
| **2** | 1,101 | 0,041 | 0,534 | 0,134 |
| **3** | 2,093 | 0,043 | 1,741 | 0,032 |
| **4** | 2,190 | 0,050 | 1,243 | 0,012 |
| **5** | NA | NA | 1,651 | 0,045 |
| **6** | 1,478 | 0,056 | 0,787 | 0,010 |
| **7** | NA | NA | 1,190 | 0,053 |
| **8** | 3,006 | 0,109 | 1,261 | 0,026 |
| **9** | 0,774 | 0,040 | 0,546 | 0,019 |
| **10** | 1,516 | 0,034 | 1,142 | 0,014 |
| **11** | 1,698 | 0,048 | 1,032 | 0,013 |
| **12** | 1,330 | 0,024 | NA | NA |
| **13** | 2,130 | 0,069 | 1,287 | 0,011 |
|  | **K1(E-)** | **SD K1(E-)** | **K1(E+)** | **SD K1(E+)** |
| **1** | NA | NA | 0,764 | 1,109 |
| **2** | 0,267 | 0,053 | 0,914 | 1,249 |
| **3** | 0,417 | 0,020 | 0,618 | 0,036 |
| **4** | 0,633 | 0,066 | 0,462 | 0,068 |
| **5** | NA | NA | 0,457 | 0,074 |
| **6** | 0,228 | 0,016 | 0,279 | 0,047 |
| **7** | NA | NA | 0,277 | 0,130 |
| **8** | 0,366 | 0,034 | 0,485 | 0,047 |
| **9** | 0,279 | 0,051 | 0,594 | 0,124 |
| **10** | 0,500 | 0,038 | 0,463 | 0,033 |
| **11** | 1,465 | 0,080 | 0,846 | 0,096 |
| **12** | 0,579 | 0,092 | NA | NA |
| **13** | 0,582 | 0,036 | 0,704 | 0,039 |

**Abbreviations:** (E+) = with erlotinib therapy, (E-) = without erlotinib therapy, V_T_ = volume of distribution, K1 = influx rate constant, SD = standard deviation, NA = not available.

## Supplementary Table S4. Tumor [^15^O]H_2_O flow values (unitless)

| **Pt nr** | **K1(E-)** | **SD K1(E-)** | **K1(E+)** | **SD K1(E+)** |
| --- | --- | --- | --- | --- |
| **1** | NA | NA | 0,763 | 0,034 |
| **2** | 0,503 | 0,038 | 0,625 | 0,099 |
| **3** | 0,553 | 0,017 | NA | NA |
| **4** | 0,208 | 0,011 | 0,329 | 0,013 |
| **5** | NA | NA | NA | NA |
| **6** | 0,409 | 0,021 | 0,320 | 0,011 |
| **7** | NA | NA | 0,354 | 0,021 |
| **8** | 0,322 | 0,029 | 0,276 | 0,032 |
| **9** | NA | NA | 0,427 | 0,055 |
| **10** | NA | NA | NA | NA |
| **11** | 1,486 | 0,071 | NA | NA |
| **12** | 0,760 | 0,066 | NA | NA |
| **13** | 0,732 | 0,026 | 0,710 | 0,021 |

**Abbreviations:** (E+) = with erlotinib therapy, (E-) = without erlotinib therapy, K1 = influx rate constant or TBF (tumor blood flow), SD = standard deviation.

## Supplementary Table S5. SUV and TBR values (unitless)

|  | **Time interval 40-50 min post-injection** | | | | **Time interval 50-60 min post-injection** | | | |
| --- | --- | --- | --- | --- | --- | --- | --- | --- |
|  | **Arterial samples** | | **Venous samples** | | **Arterial samples** | | **Venous samples** | |
|  | **SUV** | | | | | | | |
| **Pt Nr** | **E-** | **E+** | **E-** | **E+** | **E-** | **E+** | **E-** | **E+** |
| **1** | NA | 6,28 | NA | 6,28 | NA | 5,82 | NA | 5,82 |
| **2** | 4,35 | 3,07 | 4,35 | NA | 4,38 | 2,78 | 4,38 | NA |
| **3** | 5,89 | 5,41 | 5,89 | 5,41 | 5,59 | 5,03 | 5,59 | 5,03 |
| **4** | 3,58 | 6,19 | 3,58 | 6,19 | 3,48 | 5,42 | 3,48 | 5,42 |
| **5** | NA | 6,16 | NA | 6,16 | NA | 6,13 | NA | 6,13 |
| **6** | 4,25 | 5,37 | 4,25 | 5,37 | 4,40 | 4,59 | 4,40 | 4,59 |
| **7** | NA | 11,01 | NA | 11,01 | NA | 10,26 | NA | 10,26 |
| **8** | 8,38 | 7,23 | 8,38 | 7,23 | 8,16 | 6,75 | 8,16 | 6,75 |
| **9** | 5,26 | 5,68 | 5,26 | 5,68 | 5,25 | 5,20 | 5,25 | 5,20 |
| **10** | 5,16 | 6,85 | 5,16 | 6,85 | 4,87 | 5,84 | 4,87 | 5,84 |
| **11** | 5,91 | 10,85 | 5,91 | 10,85 | 5,01 | 10,16 | 5,01 | 10,16 |
| **12** | 5,54 | NA | 5,54 | NA | 5,34 | NA | 5,34 | NA |
| **13** | 8,10 | 7,41 | 8,10 | 7,41 | 7,56 | 6,96 | 7,56 | 6,96 |
|  | **TBR** | | | | | | | |
| **1** | NA | 0,92 | NA | 0,86 | NA | 0,95 | NA | 0,88 |
| **2** | 1,12 | 0,59 | 0,92 | NA | 1,23 | 0,55 | 1,02 | NA |
| **3** | 1,97 | 1,66 | 1,52 | 1,37 | 2,13 | 1,58 | 1,64 | 1,30 |
| **4** | 2,33 | 1,30 | 1,46 | 1,08 | 2,40 | 1,20 | 1,50 | 0,99 |
| **5** | NA | 1,55 | NA | 1,50 | NA | 1,78 | NA | 1,72 |
| **6** | 1,33 | 0,85 | 0,90 | 0,83 | 1,36 | 0,79 | 0,91 | 0,77 |
| **7** | NA | 1,23 | NA | 1,23 | NA | 1,33 | NA | 1,33 |
| **8** | 2,42 | 1,27 | 2,07 | 1,15 | 2,54 | 1,27 | 2,17 | 1,15 |
| **9** | 0,89 | 0,70 | 0,73 | 0,69 | 0,97 | 0,65 | 0,80 | 0,64 |
| **10** | 1,44 | 1,16 | 0,94 | 0,98 | 1,61 | 1,06 | 1,06 | 0,90 |
| **11** | 1,60 | 1,24 | 1,33 | 1,29 | 1,67 | 1,28 | 1,38 | 1,33 |
| **12** | 1,45 | NA | 1,20 | NA | 1,30 | NA | 1,07 | NA |
| **13** | 2,20 | 1,35 | 1,91 | 1,11 | 2,11 | 1,41 | 1,84 | 1,16 |

**Abbreviations:** SUV = standardized uptake value, TBR = tumor-to-blood ratio, (E+) = with erlotinib therapy, (E-) = without erlotinib therapy, NA = not available.
